# Thermophoretic Micron-Scale Devices: Practical Approach and Review

**DOI:** 10.3390/e22090950

**Published:** 2020-08-28

**Authors:** Namkyu Lee, Simone Wiegand

**Affiliations:** 1 Institute of Biological Information Processing (IBI-4: Biomacromolecular Systems and Processes) & JARA-SOFT, Forschungszentrum Jülich GmbH, D-52428 Jülich, Germany; n.lee@fz-juelich.de; 2 Department für Chemie—Physikalische Chemie, Universität zu Köln, 50939 Cologne, Germany

**Keywords:** microfluidic, thermophoresis, thermodiffusion, temperature gradients, temperature measurements

## Abstract

In recent years, there has been increasing interest in the development of micron-scale devices utilizing thermal gradients to manipulate molecules and colloids, and to measure their thermophoretic properties quantitatively. Various devices have been realized, such as on-chip implements, micro-thermogravitational columns and other micron-scale thermophoretic cells. The advantage of the miniaturized devices lies in the reduced sample volume. Often, a direct observation of particles using various microscopic techniques is possible. On the other hand, the small dimensions lead to some technical problems, such as a precise temperature measurement on small length scale with high spatial resolution. In this review, we will focus on the “state of the art” thermophoretic micron-scale devices, covering various aspects such as generating temperature gradients, temperature measurement, and the analysis of the current micron-scale devices. We want to give researchers an orientation for their development of thermophoretic micron-scale devices for biological, chemical, analytical, and medical applications.

## 1. Introduction

Since the 1990’s, there has been a dramatic increase of micron-scale devices in biology, chemistry, analytics, and medical applications. Various technical and scientific applications have been summarized in numerous reviews [1,2,3,4,5,6,7,8,9,10,11,12,13,14,15,16]. Some of the main advantages are the low volume and the short time for analysis. On the other hand, the miniaturization requires an increased sensitivity and other technical improvements to obtain reliable and serious data sets.

Thermophoresis is a phenomenon in which the movement of particles is driven by a temperature gradient [17]. Phenomenologically, the net mass flux *j* caused by thermophoresis is expressed as [18]

(1) $$j=-\rho D\nabla c-\rho c\left(1-c\right)D_{T}\nabla T$$

assuming a one-dimensional concentration and temperature gradient ∇c and ∇T with density ρ, diffusion coefficient *D*, and thermal diffusion coefficient $D_{T}$. In the steady state (j=0) the Soret coefficient, $S_{T}$, is defined as

(2) $$S_{T}\equiv \frac{D_{T}}{D}=-\frac{1}{c(1-c)}\frac{\Delta c}{\Delta T}$$

Thermophoresis or thermodiffusion is one of the unsolved puzzles in chemical physics. The Soret coefficient depends on many properties, such as mass, size, shape, pH, temperature, hydrophilicity, and ionic strength, but so far there exists no microscopic theory to predict $S_{T}$. In the past, the main focus was on the separation of crude oil and other non-polar solvents available in large amounts [19,20,21,22]. At present the focus has been shifted towards biological compounds. This led to the development of new methods that require small sample volume supporting the limited availability of biological samples of interest, such as proteins and ligands. There are two main application routes: on one hand, thermophoresis is used in combination with convective flow to accumulate and replicate biomolecules [23,24,25,26,27], and on the other hand, thermophoresis is applied as analytical tool to monitor binding reactions of biomolecules, such as between proteins and ligands [28,29,30,31,32,33]. The working horse in this field is the commercially available microscale thermophoresis (MST), which is used to determine the dissociation constant $K_{d}$, giving access to the change in Gibbs free energy. This instrument is not designed to determine the thermophoretic properties quantitatively, but helps in the search of pharmaceutical drugs [25]. This also shines some light on disease’s mechanism in medical applications such as influenza [34], Alzheimer [35] and corona [36]. It would be desirable to develop methods that provide quantitative thermophoretic values, which can be used to develop theories to explain conformational transitions and hydration layer changes during the protein-ligand binding process in order to obtain a more fundamental understanding [32,33]. A deeper understanding from the theoretical side will certainly help to optimize the search for suitable pharmaceutical compounds.

There have been several approaches to miniaturize thermophoretic cells [27,37,38,39,40,41,42,43,44,45,46]. Many instruments using laser light are operated in combination with microscopes, while others use the deflection of laser beams or interference due to refractive index contrast [38,41,47]. Some of the devices solely apply temperature gradients to manipulate particles for separation or trapping without measuring thermophoretic properties quantitatively [40,44,48,49,50,51,52]. There are different ways to create temperature gradients by using a laser beam, resistive heating, and channels at different temperatures. One objective is the maximization of the temperature gradient without the inset of free convection in the thermophoretic micron-scale devices. Another issue is the local temperature measurement with high spatial and time resolution. We will discuss different methods to measure the temperature, including their advantages and disadvantages.

The intention of this review is to give an overview of thermophoretic micron-scale devices already experimentally realized to measure thermophoretic properties quantitatively and to support researchers in their research to find the most promising configuration for their biological, chemical, analytical, and medical applications. We introduce a simple mathematical concept to describe the heat transport before giving a literature overview on various geometries generating temperature gradients and the different experimental techniques measuring the temperature locally in the micron-scale devices. This analysis is helpful to scan several experimental configurations and develop a preliminary design. Finally, we give an overview of the existing thermophoretic micron-scale devices.

## 2. Calculations and Analysis

For the development of thermophoretic micron-scale devices, it is necessary to calculate, or at least to estimate, the expected spatial and temporal distribution of the temperature and additionally we have to ensure that no free convection occurs. All types of heat transfer phenomena, such as heat conduction, convection, and radiation, need to be considered in order to estimate the temperature distribution for a particular cell design. As a first step, it is time saving to use a simplified model to get a coarse estimate of the stationary temperature distribution for a certain design, instead of using time consuming finite element calculations. This will give us a coarse estimate of the dimensions, suitable materials in terms of heat capacity and heat conductivity. Further, we can check how much we can increase the temperature gradient before free convection sets in.

One simplified model is the thermal circuit analysis using the analogy between electrical and thermal behavior, which is based on the observation that Fourier’s equation for one dimensional heat transfer takes the same form as Ohm’s law. The thermal parameters temperature difference ΔT, heat flow *Q*, heat capacity $C_{p}$, and thermal resistance *R* are identified with the electrical parameters voltage difference ΔV, current *I*, capacitance $C^{\mathrm{el}}$, and resistance $R^{\mathrm{el}}$, respectively. We take this approach, because using the electric circuit analogy is easier than solving the differential heat equation [53]. Assuming steady-state and no heat source, the one-dimensional heat equation can be expressed as [53]:

(3) $$Q=-kA\frac{dT}{dx}=kA\frac{T_{\mathrm{high}}-T_{\mathrm{low}}}{L},$$

with the thermal conductivity *k*, the cross-sectional area *A* and the length of heat flow path *L*, respectively. For the temperature difference, ΔT follows:

(4) $$T_{\mathrm{high}}-T_{\mathrm{low}}=\Delta T=Q\frac{L}{kA}=QR_{\mathrm{cond}}$$

We identify the ratio L/kA with the thermal resistance $R_{\mathrm{cond}}$ due to conduction. We also determine the thermal resistance that is caused by convection $R_{\mathrm{conv}}$ and radiation $R_{\mathrm{rad}}$, as follows:

(5) $$\begin{array}{ccc} R_{\mathrm{conv}} & = & 1/h_{\mathrm{conv}}A\\ R_{\mathrm{rad}} & = & 1/(\varepsilon \sigma A\left(T_{s}+T_{\mathrm{surr}}\right)\left(T_{s}^{2}+T_{\mathrm{surr}}^{2}\right)) \end{array}$$

with the heat transfer coefficient $h_{\mathrm{conv}}$, surface emissivity ε, Stefan–Boltzmann constant σ, surface temperature $T_{s}$, and surrounding temperature $T_{\mathrm{surr}}$. Applying the thermal circuit analysis, we can estimate the temperature field inside the device using the material properties, geometrical factors, and some of the operating conditions (heating/cooling temperature, mass flow rate) to obtain the thermal resistances that are caused by conduction, convection, and radiation. The thermal circuit analysis is useful for a preliminary design of the thermophoretic micron-scale device. Note that it is impossible to calculate the temperature field and its time dependence for a complex geometry. In this crude thermal circuit model, the heat loss during the experiment is not considered.

From Equation (3), we learn that the temperature measurement should be as close as possible to the sample channel and that tolerances of the fabrication wall thickness will influence the temperature gradient. Because of the small dimensions of micron-scale devices, the production tolerance is often a few percent of *L*, so that variation of temperature and concentration gradient vary by the same percentage. This factors can be studied using the simple model, but further improvement can only be achieved with finite element methods. Performing those calculations, we are facing two difficulties. First, the geometrical representation in the model assumes ideal smooth surfaces, which cannot be often realized experimentally. For instance, if the wall thickness varies a few percent, this can lead to substantial changes in the temperature field. The second difficulty is the knowledge of the heat transfer coefficient $h_{\mathrm{conv}}$, which changes with the flow pattern, temperature, and environmental conditions. The surface roughness significantly affects the heat transfer coefficient, so that the numerical calculations typically deviate from the experimental values [53], but it is difficult to quantify these deviations. For instance, the influence of the surface roughness on the heat transfer coefficient $h_{\mathrm{conv}}$ increases with decreasing channel size [54]. Therefore, it is useful to match $h_{\mathrm{conv}}$ with the experimentally determined value to identify the relative changes due to systematic variation of the starting geometry.

## 3. How to Generate Temperature Gradients

In the following, we will briefly discuss the different possibilities to produce temperature gradients, such as diffusion cells with Peltier elements, thermograviational micro columns, thermostated channels, resistive heating, and optical heating. Finally, we will discuss the advantages and limitations of the various configurations with respect to various applications.

**Figure 1:**
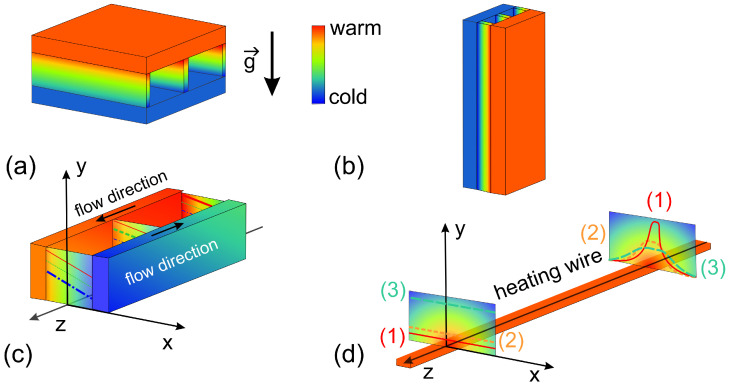
Definition of the used coordinate systems and typical configurations realized in thermophoretic micron-scale devices: (**a**) thermodiffusion cell with the temperature gradient parallel to gravitational field [55,56], (**b**) thermogravitational column configuration with a convective flow [41,47,58], (**c**) flow channel configuration [43,45,59] and (**d**) a heating wire configuration with a decrease in temperature with increasing distance from the wire [60].

Figure 1 defines the coordinate systems and compares various geometries of thermophoretic micron-scale devices. Figure 1a shows a configuration used in thermodiffusion cells [55,56,57] with the temperature gradient parallel to the gravitational field. If the cell is heated from above and the heavier components enriches in the cold region, this configuration can be operated convection free. An example of this geometry will discussed in detail in Section 5.1.1. A thermogravitational micro column (TGμC) configuration is displayed in Figure 1b. Herein, the gravitational field is perpendicular to the temperature gradient, so that the convective flow enhances the separation of the components [41,47,58]. The design details of a TGμC will discussed in Section 5.1.1. Alternatively, heating and cooling channels can be used with a counter flow (cf. Figure 1c), so that the temperature gradient remains constant along the measurement channel, but a change of the average temperature in *z*-direction cannot usually be avoided [43,45,59]. An example of a channel geometry will be given in Section 5.2.1. In a heating wire configuration [60] (cf. Figure 1b), the temperature will be constant along the wire (*z*-direction), but temperature and temperature gradient will decrease in *x*- and *y*-direction. Note that, for an ideal scenario, the isothermal planes are cylindrical surfaces around the heated wire, which lead to a two-dimensional (2D)-temperature profile in the *x*-*z*-plane complicating the thermophoretic analysis. In reality, due to the heat loss to the surrounding environment, the isothermal planes will deviate from a simple cylindrical shape. In Section 5.1.2, we will present a micro-scale device with periodic resistive heating. As in the case of the thermogravitational column also in the configurations displayed in Figure 1c,d, free convection might occur, so that the results for different temperature differences ΔT need to be compared. Performing a measurement series, the obtained results can be extrapolated to ΔT=0 to obtain a good estimate for a convection free result.

Beside the configurations displayed in Figure 1, which use thermostated walls, channels, or wires, also optical heating is implemented [23]. Typically, a laser beam, which is absorbed by the fluid in the sample channel, is focused into the sample cell with a typical channel height of 10 μm. Note that, also in this configuration, convection processes can occur and they need to be considered carefully.

The threshold of natural convection inside a cell is expressed with the non-dimensional Rayleigh number, $Ra=\beta g▵Th^{3}/\left(\nu \alpha_{solvent}\right)$ [53], with the compressibility of solvent β, the gravitational acceleration *g*, the cell height *h*, the kinematic viscosity ν, and the thermal diffusivity of solvent $\alpha_{solvent}$, respectively. The Rayleigh number should be below 1700 [61,62] in order to prevent natural convection.

### 3.1. Resistive Heating

Resistive heating, also called Joule heating, uses the resistance in an electrical conductor to convert electricity into heat. Based on Ohm’s law, the magnitude of resistive heating $Q_{\mathrm{heating}}$ relates to the potential difference ΔV across the resistance $R^{\mathrm{el}}$ and the current *I* and by [63]:

(6) $$Q_{\mathrm{heating}}=I\Delta V=I^{2}R^{\mathrm{el}}$$

if the system does not perform mechanical or chemical work. In recent years, there have been numerous devices developed that use resistive heating [39,64,65,66,67,68,69,70] to provide a homogeneous temperature environment or to generate temperature gradients. Table 1 summarizes the dimensions, the materials, the power consumption, the accessible temperature range, the resistance, and the induced maximum temperature gradient of the heater. The width and length of resistive heaters cover 0.02–5 mm and 0.02–40 mm, respectively, indicating a wide range of dimensions and high flexibility of resistive heating for local heating or larger areas. For precise local heating within the order of 10 μm, cleanroom facilities are needed, which limits the materials to conducting metals, such as platinum [64,65,66] and gold [70]. Larger structures, such as channels of several millimeters, might be filled with a liquid conducting epoxy of lead alloys [39,67,69], which can be prepared in a conventional machine shop and lab.

Using resistive heating, a temperature range between 23 ${}^{\circ }$C [39,67] and 96 ${}^{\circ }$C [64] can be covered, so that various biological and colloidal systems can be studied. The maximum temperature gradients lie between 0.2 × $10^{5}$ K/m [69] and 6 × $10^{5}$ K/m [70]. Note that the temperature gradient is, in general, position-dependent and decays exponentially with increasing distance from the heater. Vigolo et al. [39,67,69] achieved only a lower temperature gradient, because in their experiment, the measurement channel is separated by a 100μm thick wall of poly(dimethylsiloxane) (PDMS) with a low thermal conductivity of k=0.1 W/mK. Contrary to this, Tsuji et al. [70] obtained a higher temperature gradient because they reduced the gap to 0.2–1 μm. This can be achieved by implementing the heaters directly into the microfluidic device, so that there is only a thin passivation layer between the heater and the sample channel [64,68,70]. Thus, reducing the gap width leads to higher temperature gradients in the micron-scale device. Note that, in the experiment, the achievable temperature gradient might be lower than the calculated gradient due to additional heat dissipation inside the gap.

One disadvantage of using resistive heating is an increase in the overall average temperature, although the environment acts as heat sink. This can lead to systematic errors, if the measured quantities depend strongly on temperature and in some cases it might cause melting of the target materials. This is especially a problem for biological samples, which denature above a certain temperature. Thus, when using resistive heating, one might consider active cooling preventing destructive effects of the target materials.

Furthermore, the applied voltage in the heating wire might cause dielectrophoresis. This is the motion of a neutral particle caused by polarization effects in a non-uniform electric field [71]. The dielectrophoretic force *F* acting on the target materials is proportional to $\nabla E^{2}$ [71]. A high voltage drop induces an increase in the dielectrophoretic force acting on the target materials and interfering with the thermophoretic motion [72,73,74]. Close to the edge of the wire, stronger electric field gradients are expected [75]. For this reason, the experimental configurations should be carefully analyzed if resistive heating is used to generate temperature gradients, in order to avoid systematic deviations of the thermophoretic properties that are caused by additional forces.

Manufacturing electric circuits on the micro- and nano-scale using cleanroom facilities is expensive. Typically, precious metals, such as gold and platinum, are used and each fabrication step costs 20–50 USD per step.

### 3.2. Channels

Another way to generate temperature gradients is the use of cooling and heating channels in micron-scale devices. In the ideal case, one-dimensional (1D) heat transport describes the heat flow from a hot to a cold channel can be described by Equation (3). If the dimensions and thermophysical properties are known the temperature profile between the cold and hot channel can be estimated. The micron-scale devices using channels do not require clean-room facilities, as it is required for lithographical fabrication on nano-size wires and has been realized in various labs [41,43,45,47,55,56,58,59,61].

Table 2 gives an overview of typical sample volumes, gap thicknesses (between sample and channel), contact material, cooling/heating temperature and temperature gradients in the micron-scale devices using heating and cooling channels. In contrast to resistive heating, the sample is not only separated by a thin passivation layer from the heater, but by a thin wall. In order to know the temperature in the sample channel, the heat conduction through the separating wall needs to be considered. Note that, the temperature will change along the channel due to heat dissipation. Typically, there will be an increase and decrease in the cooling and heating channel, respectively. This will lead to a three-dimensional (3D) temperature profile. Wide channel cross sections of the order of mm${}^{2}$ and large flow rates of the order of mL/min. are preferable in order to minimize the temperature loss along the channel.

In the majority of the devices [41,43,45,47,55,56,58,59], water is directly used as thermostating fluid or as heat sink fluid, if Peltier elements are used [61]. So far, the operating temperatures are located in the liquid state of water. Typically, the low temperature was between 5 [43] to 35 ${}^{\circ }$C [55], and the high temperature between 20 [56] to 80 ${}^{\circ }$C [43]. In general, the temperature range can be expanded using other thermostating liquids.

While resistive heating works against the ambient temperature as a heat sink, in a channel device, the cold and the hot temperature can be controlled independently. Therefore, it is possible to work at the same average temperature by decreasing and increasing *T* by the same ΔT. Another advantage is the absence of dielectrophoresis effects, which can occur if resistive heating is used.

The choice of material embedding the cooling/heating channel will influence the temperature in the measurement channel. A material with a high thermal conductivity (k>30 W/mK) should be preferred in order to reduce the temperature change across the gap. For this reason, materials such as copper (k=401 W/mK), sapphire (k=46 W/mK) [41,47,55,56], aluminum (k=237 W/mK) [58] and stainless steel (k=13.4 W/mK) [43,45,59] are used. However, except for sapphire, most materials with a high thermal conductivity are not transparent for visible light and, therefore, they are not suitable for optical detection. Typically, transparent materials have a lower thermal conductivity (k<10 W/mK), which has, on the other hand, the advantage that heat dissipation to the environment is low, which can lead to higher temperature gradients. Nevertheless, when compared to resistive heating, the generated temperature gradients in the channel configuration are typically lower. For example, the maximum temperature gradient in a channel geometry [56] is a factor of four smaller than the gradient achieved with resistive heating [70]. The reason is the larger gap of several tenth micrometer between the heating/cooling channel as compared to the thickness of the passivation layer of several hundreds of nanometers in the case of resistive heating. This means that the thermal resistance is two orders of magnitude higher, causing the lower temperature gradient. The final choice of material depends on the requirements of the experimental design.

In general, glasses or inert crystalline materials are preferred to polymeric materials, which more easily get deformed if they are exposed to a temperature gradient. Recently, it was observed, that polydimethylsiloxane (PDMS) microfluidic channels filled with water lose their transparency upon exposure to a temperature gradient [46]. The authors hypothesize a thermophoretic process in the PDMS, so that low molecular weight PDMS chains are released from the PDMS wall entering the aqueous channel.

### 3.3. Optical Heating

Optical heating is not limited to micron-scale devices and it has already been used for decades in thermal lens experiments [79,80]. Typically, a focused laser beam is used and the light intensity is either absorbed by the solution or by suspended particles so that the temperature increases locally in the solution. In the recent years, new applications of optically generated temperature gradients in micron-scale devices have been introduced: first the so-called microscale thermophoresis (MST), which monitors fluorescently labelled biomolecules in a temperature gradient generated by an infrared laser, which is absorbed by water [23,25,40]. Secondly, various kinds of *thermophoretic traps* directing nano objects using the heat dissipated from a focused laser beam have been developed [27,42,44,81]. Sometimes, a combination of resistive heating and optical heating is used the trap colloidal particles or living cells [82].

**Figure 2:**
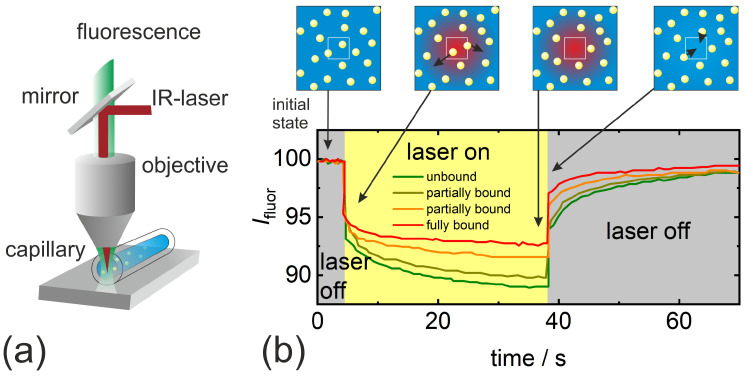
(**a**) Sketch of the microscale thermophoresis (MST) set-up (**b**) temporal dependence of the fluorescence intensity, when a heating laser is switched on and off, the fluorescently labelled particles accumulate in the cold region. Schematic temporal fluorescence intensity scans for different concentrations of the ligand molecule.

MST [25,28,40] utilizes the thermophoresis effect to monitor biochemical reactions. The basic principle is displayed in Figure 2. The device consists of an epifluorescence microscope with an additional IR-laser, which is only used to generate a temperature gradient due to strong absorption by water inside a capillary containing a solution of fluorescent molecules. The fluorescent light is observed as function of time through the same objective as the infrared laser (cf. Figure 2a). In the sketched cartoon (cf. Figure 2b), the fluorescently labelled particles leave the heated area, so that the fluorescence intensity decays towards a plateau value, which monotonically increases when the fluorescently labelled particles bind to a small ligand molecule. The plateau value with the ligand concentration determines the equilibrium binding constant of the reaction. Most of the time, only relative changes are observed, only in a few fundamental studies, the Soret coefficient has been determined by relating the established concentration gradient with the temperature gradient [23,83,84]. In these studies, the local temperature has been derived from temperature dependent fluorescence intensity (cf. Section 4.2.1). The typical applied temperature gradients are in the order of $10^{5}$ K/m.

In the *thermophoretic traps*, the local temperatures are usually not measured. The purpose of these devices is the movement of small nano objects, which are too small for optical tweezers and could only be moved with an anti-Brownian electrokinetic trap (ABEL) [85,86] or a Paul trap [75,87]. The advantage of the thermal trapping (generated using an absorbed laser beam) when compared to those traps is the free manipulation of small particles in solution without the need of rigid metal nanostructures.

Table 3 gives an overview of various methods to generate temperature gradients and summarizes advantages and limitations. Note, that another source of error can be the temperature distribution itself. It is obvious that optical heating leads to a three-dimensional (3D)-temperature distribution, but as illustrated in Figure 1 the channel configuration also needs to be applied with care to ensure a 1D-temperature profile. In the case of a resistive heating wire, we do have a 1D-temperature profile only perpendicular above the wire. In all other directions, the temperature varies in three dimensions, which complicates the analysis.

## 4. Temperature Measurements

The development of thermophoretic micron-scale devices requires the precise measurement of temperatures with a high spatial resolution of O (0.1–1 μm), because a temperature measurement with an accuracy of 0.1 K are causing already a 10% error of a Δ*T* of 1 K. An overview about temperature measurements in devices on the micro- and nano-scale are given in two recent reviews [88,89].

In Table 4, we summarize several contact and non-contact methods that are used in thermophoretic micron-scale devices. The contact methods use a resistive temperature detector (RTD) or thermocouple directly implemented in the measuring device close to the sample. The non-contact methods utilize optical detection, so that the measurement channel needs to be transparent for the detecting wavelength. Fluorescence thermometry uses the temperature dependence of the quantum efficiency, infrared thermometry measures the radiative flux and laser interferometry monitors the refractive index change with temperature. All three non-contact methods provide spatial information about the temperature distribution in the measurement channel and are not limited to a single measurement point. In the following, we will discuss the temperature accuracy and the spatial resolution of the various measurement systems and their applicability to thermophoretic devices.

### 4.1. Contact Methods

#### 4.1.1. Resistance Temperature Detector

*Measurement principle.* Resistance temperature detectors (RTD) use the temperature dependence of the electrical resistance. The physical origin is that the movements of the atoms become greater with increasing temperature, so that their motion starts to interfere more strongly with the electron flow [63]. Note that, the resistivity of metals usually increases almost linearly with temperature in the operating temperature of thermophoretic micron-scale devices. After calibrating the RTD, the temperature can be calculated from the measured electrical resistance $R^{\mathrm{el}}$. Often, platinum is used in RTDs since it is relatively free from corrosive effects as compared to other materials, such as copper, nickel, or platinum alloys.

*Specifications.* The main reason using RTDs is the good accuracy of the order of 0.1 K. Once the RTD has been incorporated into the micron-scale device, the actual temperature measurement is rather simple by means of a multimeter. Determining the resistance does not require transparency, therefore, we can use all materials with an appropriate dielectric layer as substrate. However, RTDs have several disadvantages: The spatial resolution is poor, even if micro-nano fabrication techniques help to achieve smaller length scales, the resolution is limited to the order of 10 μm. Furthermore, a RTD only measures at a single point and cannot capture the spatial distribution in the entire device. Therefore, a linear temperature distribution has to be assumed for extracting the temperature gradient to determine the thermophoretic properties. Even if special care is taken to ensure linear temperature profiles and avoid heat bridges, the obtained data should be confirmed at several temperatures using validated methods [97].

*Application examples.* RTDs are used in several devices from the micron-scale [98,99] to the macro-scale [100,101]. Thermophoretic devices using RTDs are typically positioned at the edge of heating/cooling channel/plate. The temperature gradient is calculated based on the 1D-equation for heat conduction [39,58,90,91,92]. Note that the beam deflection set-up of Königer et al. and the Soret cell on the international space station have been validated with the benchmark compounds [57,97,102].

#### 4.1.2. Thermocouple

*Measurement principle.* A thermocouple uses a thermoelectric effect, the so-called Seebeck effect. At the junction of two different electrically conducting materials, one can measure a small potential difference between the ends of the materials, which depends on the temperature at the junction [103,104]. If the correlation between the electrical potential and temperature is known, then it is possible to determine the temperature at the junction by measuring the voltage. As this is a simple configuration, it has been implemented in a number of thermophoretic devices close to heating and cooling parts [55,56,61,69].

*Specifications.* The accuracy of a thermocouple of O(0.01K) is one order of magnitude better than for RTDs and the actual voltage measurement is straightforward. Additionally, using cleanroom facilities, it is possible to reduce the size of the junction (measuring point) to 0.1 μm [105,106,107]. However, the voltage level is rather low, so that external electric fields can induce some noise. Depending on the actual configuration, it might be necessary to shield the fields. The thermocouple can only measure the temperature at a single point close to the actual measurement channel, therefore, the temperature distribution in the device needs to be calculated assuming 1D heat flow between measurement points, as in the case of the RTDs.

*Application examples.* There are several thermophoretic micron-scale device, which use commercial thermocouples for temperature measurements [55,56,61,69]. Vigolo et al. placed a thermocouple in direct contact with a 170 μm thick cover slip below the heater and the cooler on each side of the channel [39]. In other experiments, the thermocouples are placed on the heating and cooling plate [55,56] or inside a bore hole of a copper plate [61]. All of the setups suffer of systematic temperature difference between the measurement point and sample channel. Additionally, temperature profiles are only calculated using the 1D heat equation and neglect systematic temperature deviations due to heat bridges across the channel.

In summary, the contact methods, such as RTD and thermocouple, have a good and excellent accuracy, respectively. Performing the actual measurement is, in both cases, simple and fast. However, due to the single point measurement, special care needs to be taken for ensuring linear temperature profiles in the measurement cell for all temperatures. Especially, if the average temperature deviates more than 20 K from the temperature in the lab, heat bridges might cause deviations from linearity [53].

### 4.2. Non-Contact Methods

#### 4.2.1. Fluorescence Thermometry

**Figure 3:**
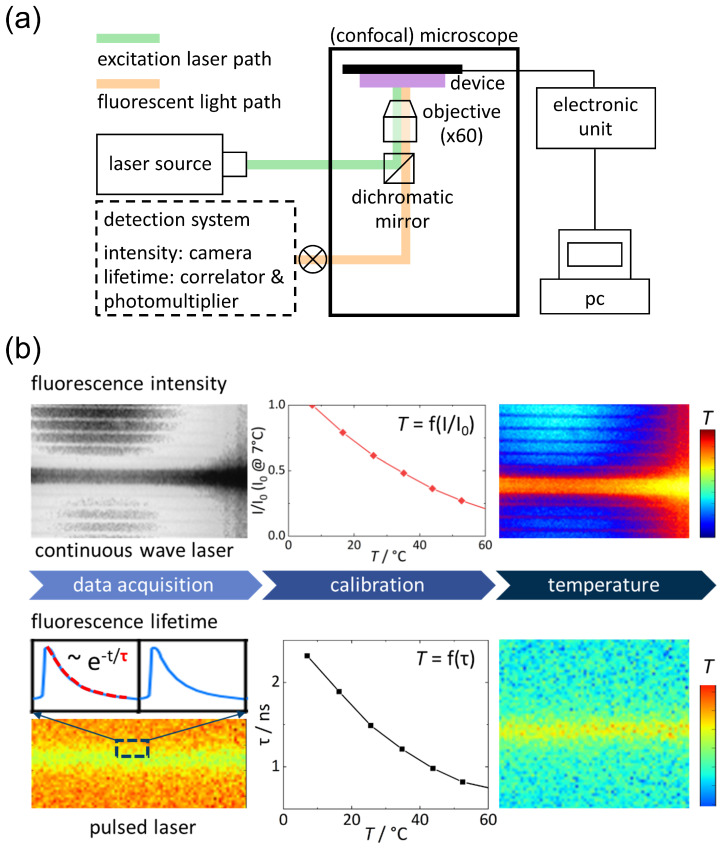
(**a**) Sketch of a fluorescence thermometry set-up for a thermophoretic chip (**b**) Procedures to convert the fluorescence intensity (**top**) and fluorescence lifetime (**bottom**) into a temperature.

*Measurement principle.* In fluorescence thermometry, a fluorophore is used to measure the temperature of a fluid or at a surface. When the fluorescent dye molecules are excited by an external light source such as a laser, the excited molecules return after a characteristic time to the ground state emitting fluorescent light of a specific wavelength (cf. Figure 3). If the fluorescence intensity and lifetime of the dye depend strongly on temperature, both properties can be used to monitor the temperature in micron-scale devices. The sample cell is then filled with a known concentration of a suitable fluorophore in an appropriate solvent. It is useful to check concentrations and solvents that give the most stable and reliable results [108].

A typical fluorescence thermometry set-up is shown in Figure 3a. The fluorescence intensity can be measured with an ordinary fluorescence microscope, a continuous wave laser and a sensitive camera without or disabled gain control. Applying a calibration curve the fluorescence intensity image can be converted into a temperature profile (cf. top part of Figure 3b). For measuring the fluorescence lifetime, a confocal microscope, a pulsed laser, a photomultiplier, and correlator are required.As illustrated in the bottom part of of Figure 3b the fluorescence correlation function is measured for each point to get a lifetime image. This is then converted into a temperature profile applying a separately measured calibration curve. Note that the solvent affects the fluorescence lifetime [109]. Both fluorescence methods require materials that are transparent for the used wavelengths. In general, the transparent materials have a lower thermal conductivity, leading to smaller temperature gradients. As a consequence, a better signal-to-noise ratio of the temperature measurement is needed to achieve the same reliability of the data.

*Specifications.* Typically, fluorescence measurements are done under a microscope with a high resolution objective leading to a spatial resolution of O (1 μm) and a thermal accuracy of O(0.1K). Note that the thermal accuracy of O(0.1K) can only be achieved for fluorescence lifetime measurements, if the calibration is repeated several times in order to reduce the noise. In general, the fluorescence lifetime measurements are more robust than recording the fluorescence intensity [88]. The intensity measurements are affected by laser intensity fluctuations, altered by reflected light, and by photobleaching diminishing the fluorescence intensity with time. This leads to systematic errors in the temperature read-out of O(1K). There might be special conditions, such as heterogeneous biological samples, where the fluorescence thermometry using the intensity measurement might be superior to lifetime evaluation [110]. For instance, fluorescence lifetime imaging in cell nucleus and plasma [111] lead to unrealistic large temperature differences [96], which might in this particular work been caused by the fact that the calibration curves have only been determined for buffer solutions. Therefore, it is important to keep in mind that fluorescence thermometry strongly depends on the environment requiring an in-situ calibration with the same solvent, same ionic strength and identical viscosity to obtain reliable results in the experiment.

*Application examples.* Several researchers have already utilized the fluorescence intensity method with various dyes (RhB [43,45,59], BCECF [27,70,93], Ruthenium [94,95]) to observe the temperature field in a thermophoresis experiment. To our best knowledge, despite the experimental robustness, fluorescence lifetime imaging measurements have not been used in thermophoretic studies.

#### 4.2.2. Infrared Thermometry

*Measurement principle.* Infrared thermometry detects the emitted radiation of an object in the infrared. Most detectors record the intensities in a wideband between 8–14 μm, which avoids interference from atmospheric components over long paths. For special application, more expensive narrowband detectors in the short infrared wave regime 1–5 μm are also used [112]. The commercial instruments provide, in general, an image of the temperature distribution and the measurement is easier than other non-contact methods, which might require an experimental adaptation or the insertion of specific temperature sensitive tracers.

*Specifications.* The accuracy of the temperature is in the best case ±1 K and the spatial resolution of commercial instruments is at most O (15) μm. Depending on the field of view (FOV), the camera might detect radiation stemming from objects near or behind the object of interest, which will distort the temperature measurement. Therefore, it is desirable to have additional single point measurements by a RTD or a thermocouple for calibration. Furthermore, the infrared camera measures the temperature at a surface, as IR radiation is typically absorbed within a solution.

*Application examples.* Several researchers have used the infrared camera to measure the temperature in micron-scale devices [112,113]. For the thermophoretic device, the infrared camera is applied for measuring the temperature of the two plates of a micro thermogravitational column stabilized a two different temperature [41,47]. The temperature gradient inside the cell was then estimated by solving the heat equation.

#### 4.2.3. Laser Interferometry

*Measurement principle.* Laser interferometry sometimes called thermal imaging uses either quadriwave shearing interferometry (TIQSI) [81,96] or optical digital interferometry (ODI) [114,115] to measure the phase difference, when the refractive index of the solution changes with temperature. The method relies on the temperature derivative of refractive index ${\left(\partial n/\partial T\right)}_{c,p}$ to convert a phase change into a temperature change. Note that this technique depends on a change of the phase and cannot be used to determine a steady state distribution. Furthermore, we have to ensure that ${\left(\partial n/\partial c\right)}_{p,T}=0$, which is only true for one component systems, in order to relate the observed phase with a temperature change. If the system shows heterogeneities or consists of more than one component, the method is not applicable.

*Specifications.* Using a CCD camera, laser interferometry provides a temperature profile with a high spatial resolution of the order of O (0.1 μm) with a temperature resolution of O(1K) [81,96]. Sometimes, it is slightly better, such as ∼0.4 K [96], but not of the $O\;(10^{-6}\;K)$, as it has been erroneously listed in Table 2 in the review by Kim et al. [88].

*Application examples.* Mialdun and Shevtsova developed optical digital interferometry (ODI) to measure thermodiffusive properties [114,115,116]. As the first step, the temperature profile in a Soret cell has been characterized using a pure liquid. This characterization is essential to obtain reliable data. In general, the assumption of a linear temperature profile between the hot top and the cold bottom plate is not fulfilled. Lateral heat fluxes are observed, probably due to different heat conductivities of materials in contact and the heat fluxes through walls in contact with surrounding air at ambient temperature [114,115] leading to deviation of O∼0.5 K close to the thermostated plates. Nevertheless, a careful analysis reproduces the thermophoretic properties of the benchmark systems within 3% [116].

## 5. Micron-Scale Devices

### 5.1. Validate Devices

In the following section, we present three validated thermophoretic micron-scale set-ups. We discuss a micron-scale beam deflection [37,77,78], periodic resistive heating [38] and a thermogravitation micro-column [41], which has recently been improved using digital interferometry [47]. All of the devices have been validated using the benchmark compounds [97].

#### 5.1.1. Micron-Scale Beam Deflection

**Figure 4:**
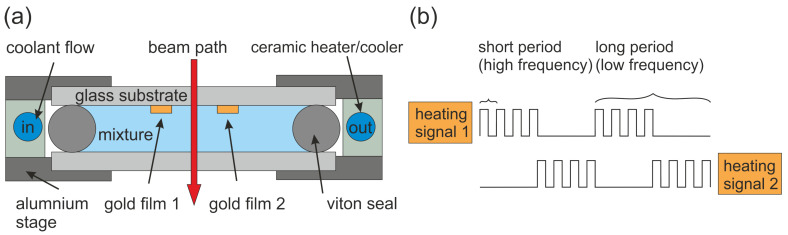
(**a**) Sketch of the micron-scale beam deflection set-up. (**b**) Alternating heating current of the two wires.

Putnam and Cahill [37,77,78] describe a micron-scale beam deflection technique to measure the Soret coefficient of fluid mixtures and particles suspensions. In contrast to a classical beam deflection with a parallel-plate geometry [57], the micron-scale device is approximately 300-times faster and it only has a cell volume between 20–70 μL. Figure 4a shows a schematic sketch of the micron-scale beam deflection set-up. Two thin gold films (≈250 nm thick, 10 μm in width, and 2 mm in length) are deposited on a glass substrate covering the liquid mixture from above to avoid convection. The fluid cell is sealed mechanically using a viton O-ring and placed in a ceramic heater for temperature control. The temperature gradient between the two gold films is probed with a laser beam traversing the cell from above, whereas the two gold films a heated alternating with a square wave electric current. For the heating period, a high frequency square wave is used to reduce the electric field effects (cf. Figure 4b). They analyzed the real and imaginary part of the deflected beam as function of the low frequency, while the high frequency was fixed to a constant value in the range of 5–6 kHz. The estimated typical temperature amplitude is of the order $\Delta T_{\mathrm{osc}}\approx 0.3$ K [37]. For larger $\Delta T_{\mathrm{osc}}\geq 0.3$ K, they observed significant discrepancies between experiment and their analytical model at extremely low modulation frequencies, f≤ 10 mHz, which they assigned to convection effects [77].

In the case of uncharged polystyrene (PS) polymers in toluene and for charged PS microspheres in deionized water, the same results were obtained for high-frequency and dc heating [37]. Additionally, they reproduced the benchmark value for a 50–50 wt% mixture of dodecane (C12H26) and 1,2,3,4-tetrahydronaphthalene (THN) in the micron-scale apparatus [77]. As mentioned before, using resistive heating can lead to dielectrophoresis in mixtures with charged constituents. Another complication is the formation of an AC current between the two gold films, which can lead to electrochemical decomposition of the gold films [117].

#### 5.1.2. Periodic Resistive Heating

**Figure 5:**
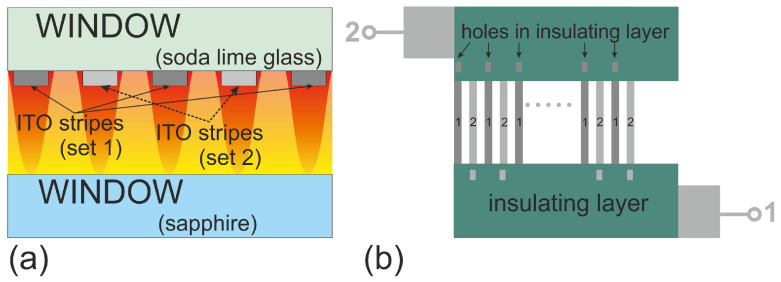
Sketch of the cell used by Hartung and Köhler [38]: (**a**) A grating of transparent conducting strips (ITO) is at the inside of one of the windows. When heated by an electric current *I* a temperature grating will build up in the sample, which decays exponentially with increasing distance from the heated stripes. (**b**) Multilayer structure of ITO strips with insulating layers.

Hartung and Köhler [38] used Joule heating to create a periodic temperature modulation, similar to transient optical grating used in thermal diffusion forced Rayleigh scattering (TDFRS) [118]. For that purpose, they used two regular cogged arrays of transparent strips of indium tin oxide (ITO) with 10 μm width and a grating period of 40 μm on one inner window of the cuvette, as shown in Figure 5. The two windows are separated by a spacer of 100 μm thickness. When heated by an electric current *I*, a temperature grating builds up in the sample, whereas the temperature decays exponentially with increasing distance from the heated stripes. Therefore, the diffraction efficiency of this grating is less than 0.2-times of the efficiency of an optical grating with the same power. Typical temperature gradients are of the order of 500 μK/μm. The complicated structure of two cogged stripe sets had been chosen in order to determine the heterodyne diffraction signal. The temperature distribution in the cell has been calculated solving the heat equations. Based on the determined temperature profiles as function of time and position, the three organic benchmark systems [97], symmetric mixtures of dodecane, isobutyl benzene, and tetralin, have been analyzed. Hartung and Köhler found that the measured Soret and diffusion coefficients were over- and underestimated by roughly 10% compared to the benchmark values, respectively. Unfortunately, the cell was not suitable for aqueous systems, which probably caused electrochemical reactions at the ITO electrodes. Those reactions might be avoidable using thin and inert protective layers.

Note that one other complication of this design is the multilayer structure. It is rather challenging to fabricate two layers of homogeneously heated stripes, which are electrically insulated.

#### 5.1.3. Thermogravitational Micro-Column

**Figure 6:**
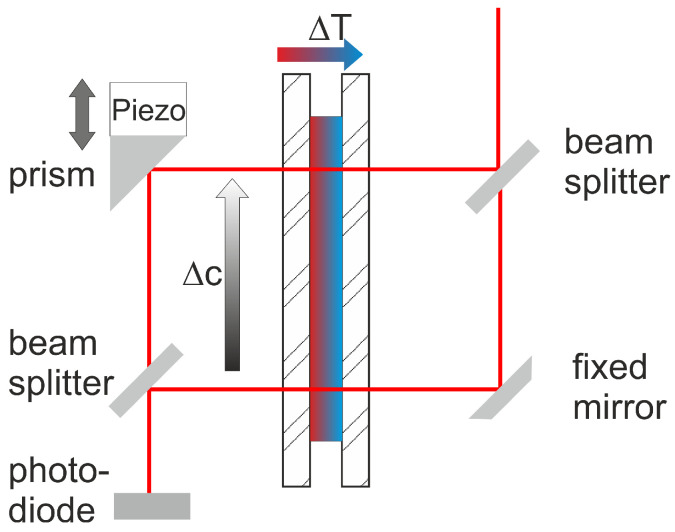
Schematic sketch of the interferometer probing the concentration difference at two different heights of the TGμC with a horizontal temperature gradient ΔT. The concentration difference results in a phase shift of the intensity signal determined by a 2π scan of the prism mounted on the piezo stack.

Several years ago, Naumann et al. [41] developed a thermogravitational micro-column (TGμC) with a cell volume of approximately 45 μL (cf. Table 2). This technique enhances the concentration difference due to a combination of thermophoretic motion and convection. Most thermogravitational columns work without optical detection, because the heated and cooled walls are made of non-transparent materials e.g., steel or brass. In this TGμC, transparent sapphire windows are used as walls (cf. Figure 6). The sapphire windows are in thermal contact with copper plates traversed by tempered water. Sapphire has a very good thermal conductivity, so that a homogenous temperature can be achieved. Having transparent walls, it is possible to measure the concentration difference between top and bottom interferometrically. The upper beam is probing the top of the μ-column and the lower beam is probing the bottom of the μ-column, as illustrated in Figure 6. From the phase difference Δϕ, the thermal diffusion coefficient $D_{T}$ can be evaluated by

(7) $$D_{T}=\frac{1}{504}\frac{\alpha g}{k^{wave}\nu }\frac{1}{c_{0}\left(1-c_{0}\right)}\frac{L_{x}^{3}}{L_{z}}{\left(\frac{\partial n}{\partial c}\right)}_{p,T}^{-1}\Delta \phi$$

with the initial mass fraction concentration $c_{0}$, the wave vector $k^{wave}$, the kinematic viscosity ν, the thermal expansion coefficient α, the vertical distance $L_{z}$ between the probing laser beams, the gap width $L_{x}$, and the gravity acceleration *g*. Note that the fluid properties are taken at the average temperature. The determined $D_{T}$ values deviated by about 5% from the benchmark-values determined for the three organic binary mixtures. This is comparable with the deviations that were found for different methods used in the benchmark [97]. The advantages of this cell are the good conditions for temperature measurements, the strong signal due to a combination of convection and thermophoresis and the homogenous temperature field.

Recently, Lapeira et al. [47] improved the detection method using two lasers with different wavelengths. Instead of measuring the phase difference between two points, they analyzed phase contrast images using a digital interferometer. They could confirm the benchmark values for all the binary mixtures within a few percent [97].

The interferometric detection is very sensitive, but this requires optical surfaces with a flatness in the order of λ/10 or λ/20. The drawback of the interferometric detection using one wavelength is the limitation to binary mixtures or two ternaries with different diffusion constants, such as colloids in a binary solvent mixture. Using the improved detection system by Lapeira et al. [47], it should be possible to investigate ternary systems, as in the case of the two color beam deflection [91]. Alternatively, it might also be interesting to combine the transparent cell with another detector/spectrometer.

### 5.2. Non-Validated Devices

Note that all new thermophoretic micron-scale devices that operate under a microscope using optical laser heating [23,39,43], a channel geometry [43,45,76] or diffusion cell have not been validated by comparing the results with the benchmark values [97]. The reason is that the modern methods using a microscope to monitor the concentration profile or to analyze the particle velocity, require colloids of larger macromolecules with fluorescent labels, while concentration changes of the benchmark systems (organic solvents) [97] cannot be monitored with these microscope methods.

#### 5.2.1. Thermophoretic Micron-Scale Channel

**Figure 7:**
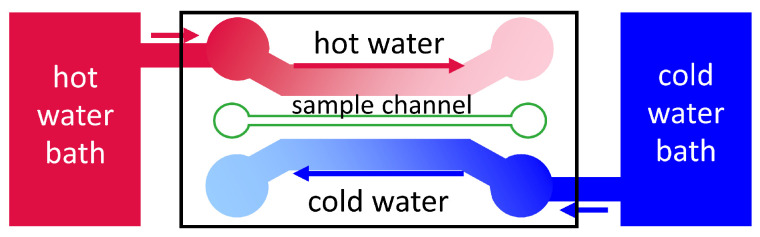
Schematic sketch of the experimental setup with a heating and a cooling channel connected to a hot and a cold water bath, respectively. The smaller sample channel contains the sample e.g., fluorescently labeled colloidal particles. The cell is operated under a fluorescence microscope with a CCD camera that was connected to a computer for data collection.

The group of Yang [43,45,59,76] developed a micron-scale channel with a cell volume of approximately 0.6 μL (cf. Table 2). As sketched in Figure 7, the sample channel lay in the middle between a heating and cooling channel connected to thermostats. The counterflow principle is applied in order to ensure a uniform transverse temperature gradient along the channel. All the channels had been milled in stainless steel and had been sealed with a transparent plastic cover, so that the cell could be monitored with an inverted fluorescence microscope. Using the temperature sensitivtiy of the fluorescence intensity of a dye (Rhodamine B) they estimated a constant transverse temperature gradient of around 1.5 × $10^{4}$ Km${}^{-1}$ and a two orders of magnitude smaller temperature gradient in the longitudinal direction. They investigated fluorescent polystyrene beads, so that the concentration distribution in the steady was obtained from an experimentally determined non-linear calibration curve of the fluorescence intensity as function of the particle concentration [43]. In the steady state according to Equation (2), the Soret coefficient $S_{T}$ can be determined by

(8) $$S_{T}=-\frac{\ln\left(\frac{c_{0}+\Delta c}{c_{0}}\right)}{T-T_{0}}=-\frac{\ln\left(1+\frac{\Delta c}{c_{0}}\right)}{\Delta T}\approx -\frac{1}{c_{0}}\frac{\Delta c}{\Delta T},$$

with the initial concentration $c_{0}$, the measured differences in concentration Δc and temperature ΔT, respectively. Note, that we used in Equation (8) the approximation ln(1+ε)≈ϵ valid for small values of ε. In a revised version of the set-up [45,59], they measured the thermophoretic velocity ${\vec{u}}_{T}$, which is related to the thermal diffusion coefficient $D_{T}$ by

(9) $${\vec{u}}_{T}=-D_{T}\nabla T.$$

In those experiments, they encountered experimental difficulties for the larger polystyrene beads, as they can only be tracked for a certain time before sedimenting due to their larger density compared to water.

In most of the studies with the channel geometry [43,45,59,76] fluorescent polystyrene beads in a diameter range between 100 nm and 5 μm have been investigated. Note that, according to the information provided by the supplier, the suspension contains a trace amount of ethylenediaminetetraacetic acid (EDTA) and an anionic surfactant that is similar to SDS as a preservative to inhibit aggregation and promote stability. In the channel studies [43], the particles were washed to remove EDTA and SDS. At 1 wt%, the washed particles with a diameter of 100 nm showed a negative Soret coefficient. There are no literature results that could be used for direct comparison. In a recent study of the same kind fluorescent polystyrene beads (same supplier) with a smaller diameter of 25 nm, a positive Soret coefficient was observed for the washed particles with a validated method [119]. This study also demonstrated that this particular system is very sensitive to the surface coverage of the colloidal particles. For instance, for the unwashed particles, a negative $S_{T}$ was obtained with a validated method [119].

#### 5.2.2. Microscope Cell

**Figure 8:**
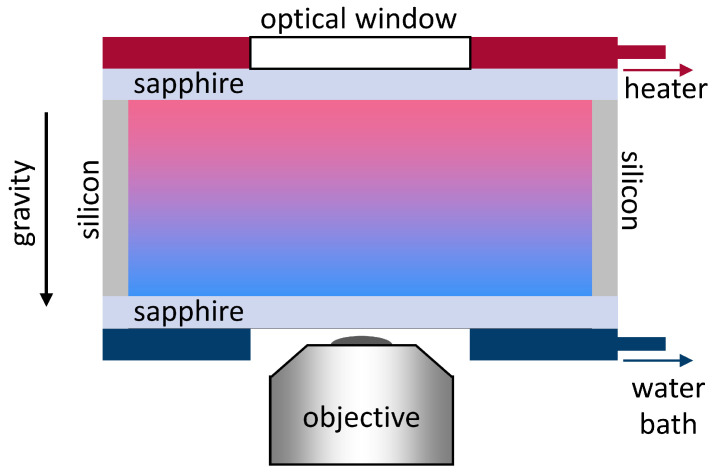
Schematic sketch of the temperature control cell. This figure is not to scale.

Talbot et al. [55] used a microscope cell (cf. Figure 8) to investigate the thermophoretic behavior of vesicles. Heating and cooling plates were controlled by Peltier elements, with a standard deviation of 0.03 K for each plate. The heating part had an optical window for illumination and the cooling part contained a center hole for an objective of an inverted microscope [120]. The cell was heated from above to avoid convection and the achieved temperature gradient ranged from 0.05–0.15 K μm${}^{-1}$. Between the heating and cooling parts, the sapphire windows were separated by silicon spacer with a thickness of 200 μm resulting in a sample volume below 240 μL (cf. Table 2).

Thermocouples were used to probe the temperature at the heating and cooling plate. The linearity of the temperature profile across the cell was confirmed by measuring the diffusion coefficient as a function of height. They assumed spherical vesicles with a diameter of 1 μm and found that the change in diffusion coefficient corresponds to the temperature dependence of the viscosity. This indirect method has a large uncertainty, especially at the large temperature gradients, since deviations from linearity were observed (cf. Figure 6 in the supplementary information of Ref. [55]). The fluorescently labeled vesicles were illuminated with a metal halide lamp or a single-color light-emitting diode. The concentration changes as a function of height was estimated from the area fraction of vesicles (white pixel/total pixel) in each frame. Using the temperature and concentration differences, the Soret coefficient can be determined using Equation (8).

The paper revealed that the Soret coefficient changed with the lipid head group (e.g., phosphatidylglycerol (PG), phosphatidylserine (PS), phosphatidic acid (PA)), while the unexposed tail did not significantly influence the Soret coefficient. Further, they observed the monotonous increase of $S_{T}$ with temperature, which is typical for aqueous systems [17]. The obtained Soret coefficients of vesicles are within the expected range, but there are no literature results to be compared with.

## 6. Conclusions

Motivated by various applications in biotechnology recently, there has been increasing interest in the development of micron-scale devices using thermophoresis. Many applications focus on binding affinities and stability of proteins, so that they are facing challenges, such as limited sample amounts and high throughput analysis stimulating the development of lab-on-chip devices. With this review, we summarize the literature; give a simplified approach to analyze temperature fields for prototypes (cf. Section 2), discuss various methods to generate temperature gradients (cf. Section 3) and measure temperature (cf. Section 4) in thermophoretic devices with high spatial resolution.

The development of a thermophoretic micron-scale devices has to be accompanied by simulations (cf. Section 2), but it often turns out that there are deviations between the simulated and the real experiment due to the unknown heat transfer coefficient, which is affected by many parameters, such as geometry (plane, cylinder, sphere, roughness), boundary conditions (adiabatic, constant heat flux/temperature), and flow conditions (laminar, turbulent). An important point to check in these simulations is the occurrence of free convection and whether this will alter the concentration profile of the compounds under investigation [23]. As it is impossible to depict all relevant parameters in the simulation, it is essential to measure the temperature distribution within the sample channel and not rely on linear interpolation between two points measured with thermocouples or thermistors.

In thermophoresis, the established concentration difference, $\Delta c=S_{T}\Delta T$, is proportional to the temperature difference ΔT, which implies that a large temperature gradient should be created in the device to obtain a good signal-to-noise ratio. However, large gradients can cause free convection, which distorts temperature and concentration fields. Accordingly, the Rayleigh number (cf. Section 3), describing the inset of convection, needs to be optimized for a given geometry in order to prevent convection. Note that decreasing the size of the measurement channel or cell reduces the Rayleigh number, so that larger gradients in micron-scale devices can be applied when compared to conventional techniques.

Precise temperature measurements are crucial to increase the accuracy of the measured Soret coefficient. The temperature measurement accuracy is of the order of 0.1 K, implying that a temperature gradient of 1–5 K/100 μm can be resolved with an accuracy of 2–10%. Additionally, uncertainties in the concentration measurement of typically 5% and a small 1% error in the measurement of the dimension lead to an uncertainty of $S_{T}$ of up to 8–16%, depending on the gradient. Larger temperature gradients lead to smaller uncertainties but too large gradients lead to free convection. Accordingly, the accuracy of infrared and laser thermometries O∼1 K leads to errors of 25% and higher. Additionally, external conditions such as the temperature in the lab influence the temperature distribution in the cell leading to a distortion of the linear temperature profile. Thus, a direct measurement inside the cell is recommended.

Because the most important application of thermophoresis is the investigation of protein-ligand binding, which generally have to be stabilized in a buffer solution, it is desirable to have an optical transparent cell, which can be operated under a fluorescence microscope. In this way, the distribution of the proteins can be determined using a fluorescent label without the buffer components contributing to the signal, as is the case with optical methods that are based on refractive index contrast [33]. To get as close as possible to the ideal case of a one-dimensional temperature profile, a channel (cf. Figure 1c) or diffusion cell (cf. Figure 1a) configuration is preferred compared to a heated wire or an optically generated temperature profile. It is to be noted that the latter is of course more flexible. Experimentally realized examples for a channel or diffusion cell operated under a microscope are discussed in Section 5.2.1 and Section 5.2.2, respectively. Both of the methods have not been validated so far (cf. Section 5.2), so we cannot decide on the basis of a literature comparison which method gives more reliable results. The diffusion cell arrangement heated from above has the advantage that there is no free convection when the denser component accumulates in the cold region. The disadvantage of this configuration is that the gradient is formed in the vertical direction in which the microscope objective has a poorer spatial resolution than in the horizontal plane [55]. In contrast, the channel arrangement is susceptible to free convection, but it has the advantage of a good spatial resolution in the horizontal plane. Furthermore, by applying different temperature gradients and extrapolating to zero, systematic errors due to free convection can be calculated. Simulations are helpful at this point, since the influence of convection depends on the compounds under investigation. Slowly diffusing solutes are much more susceptible to convection [23].

Thus, it is essential that these microscope methods are validated. This is not so easy, since macromolecules or colloids whose diameters and surface composition are statistically distributed must be used for the comparison. Especially, the reproducibility of the surface composition is critical, since these changes are particularly influencing the thermophoretic properties. Therefore, it is necessary to use particles from the same synthesis and define a unique protocol. In our opinion, the most feasible approach is the use of small fluorescence-labeled macromolecules that can also be investigated with one of the validated methods.

The final objective is to build a micron-scale device, which can be used to determine quantitative thermophoretic properties, such as the Soret and the thermal diffusion coefficient. As thermophoresis is so sensitive to changes in the hydration layer during the binding process, a deeper understanding will help to develop models and theories for protein-ligand binding to improve and accelerate the search for suitable drug compounds and other ligands.

## Figures and Tables

**Table 1:** Resistive heating: typical dimensions of the heated areas, material, power consumption, operating temperature range, resistance, maximum temperature gradient, and reference.

Dimensions (Heater)	Material	*P*/W	*T*/${}^{\circ }$C	$R^{\mathrm{el}}$/Ω	Max. GradT	Ref.
Width × Length × Height/mm${}^{3}$	Thickness/nm				K/m	
5 × 5 × 1 × $10^{-4}$	Ti/Pt (10/100)	0.55–2	90–96	-	-	[64]
2 × 40 × 0.025	Silver filled epoxy	0–1.4	23–75	6	0.32 × $10^{5}$	[39,67]
0.02 × 0.02 × 2.45 × $10^{-4}$	Cr/Pt/Cr (2/40/3)	0–11 × $10^{-3}$	80	1500	-	[68]
1 × 30 × 0.12	lead alloy MCP-96	-	-	-	0.2 × $10^{5}$	[69]
0.02 × 0.4 × 1.5 × $10^{-4}$	Au (150)	0.4	49–92	139	6 × $10^{5}$	[70]

**Table 2:** Channels: typical sizes of microchannel, gap size between cooling/heating channel and operating channel, contact materials, cooling/heating temperature, maximum temperature gradient, and reference.

Sample	Gap Thick-	Contact Material	Cooling/Heating	Max. Grad T	∇T/∇g	Principle	Ref.
Volume/μL	Ness/mm		Temp. ${}^{\circ }$C	Temp. K/m			
0.6	-	Stainless Steel	5/80	0.15$\cdot 10^{5}$	h/v	microscope	[43,45,59,76]
≪240	0.75	Cu, Sapphire	5–35/20–65	1.5$\cdot 10^{5}$	v/v	microscope	[55]
≪200	0.5	Cu, Sapphire	20/20–50	$1.55\cdot 10^{5}$	v/v	microscope	[56]
≪5	-	Cu/PMMA	25/25–45	$1.4\cdot 10^{5}$	v/v	microscope	[61]
45	1.29	Cu, Sapphire	22/27	0.16$\cdot 10^{5}$	h/v	TGC	[41,47]
940	-	Al	22.5/27.5	$0.1\cdot 10^{5}$	h/v	TGC	[58]
20–70	0.025–0.05	Au	5–80/5.3–80.3	$1.2\cdot 10^{4}$	h/v	beam deflection	[37,77,78]

**Table 3:** Advantages and limitations of resistive heating, channels, and optical heating to generate the temperature gradients in micro-scale devices.

Methods	Advantages	Limitations
resistive heating		heat sink needed
	direct implementation	temperature controlled close to cell
	large *T*-gradients possible	additional effects (e.g., dielectrophoresis)
		expensive
channels	conventional fabrication	lower temperature gradients
	heat sink not required	external thermostat required
optical heating	large *T*-gradients possible	transparent material required
	free manipulation	heat sink needed
		temperature depends on sample absorption

**Table 4:** An overview of temperature measurements in thermophoretic devices.

Measurement	Principle	Spatial Resolution	Accuracy	Domain	Reference
contact method	advantages:	good accuracy			
		simple and fast configuration of measuring system			
	limitations:	single point, assumption of linear temperature profile			
resistance	resistance	∼10 μm	∼0.1 K	point	[39,58,90,91,92]
temperature	variation				
detector	on *T*				
thermocouple	Seebeck effect	∼0.1 μm	∼0.01 K	point	[55,56,61,69]
non-contact method	advantages:	direct measure of the temperature profile			
	limitations:	transparent materials, moderate accuracy			
		calibration required			
fluorescence thermometry	temperature dependent quantum efficiency	∼1 μm	∼0.1 K	area	[27,43,45,59,70,93,94,95]
infrared thermometry	radiative flux	∼10 μm	∼1 K	area	[41,47]
laser interferometry	phase difference	∼0.1 μm	∼1 K	area	[81,96]
